# Monocortical fixation for locking plate distal screws does not impair mechanical properties in open-wedge high tibial osteotomy

**DOI:** 10.1186/s12891-021-03999-y

**Published:** 2021-02-08

**Authors:** Junya Itou, Umito Kuwashima, Masafumi Itoh, Koichi Kuroda, Yasuharu Yokoyama, Ken Okazaki

**Affiliations:** 1 Department of Orthopaedic Surgery, Tokyo Women’s Medical University, 8-1 Kawada-cho, Shinjuku-ku, Tokyo, 162-8666 Japan; 2 Olympus Terumo Biomaterials Corp., Sasazuka NA Bldg., 1-50-1 Sasazuka, Shibuya-ku, Tokyo, 151-0073 Japan

**Keywords:** Open-wedge high tibial osteotomy, Finite element analysis, Monocortical fixation

## Abstract

**Background:**

The neurovascular bundle containing the deep peroneal nerve has a potential risk of injury during open-wedge high tibial osteotomy (OWHTO), particularly due to drilling for bicortical fixation at distal screw holes. Therefore, monocortical fixation is recommended for distal fixation of a long locking plate as long as good stability is ensured. The purpose of this study was to analyse the biomechanical properties of monocortical fixation of distal locking screws for OWHTO.

**Methods:**

Three-dimensional models of bone and fixation materials simulating OWHTO were created using computed tomographic data of patients and material data of a T-shaped long locking plate and screws. Three of the four distal screws of the locking plate were chosen for a bicortical fixation or monocortical fixation procedure. In addition, loss of correction was assessed by measuring the medial proximal tibial angle (MPTA) in patients who underwent OWHTO with two bicortical and two monocortical distal fixation screws at 1 month and 1 year after surgery.

**Results:**

No significant differences in stress were observed in either the normal or osteoporotic bone model between the monocortical and bicortical fixation models, including in the area of the lateral hinge at the osteotomy site.

Furthermore, there were no significant differences in MPTA between the early post-operative period and 1-year follow-up.

**Conclusions:**

The monocortical fixation method for three distal screws of the locking plate did not worsen the mechanical properties of fixation for OWHTO using a long locking plate with four proximal and four distal screws. In actual surgery, the number of distal bicortical screws should be reduced based on the patient’s condition, taking into account the risk of lateral hinge fracture and unexpected surgical complications. Using at least two bicortical screws would be practical considering the various factors related to reduced fixing ability.

## Introduction

Open-wedge high tibial osteotomy (OWHTO) is an effective procedure for treating medial osteoarthritis of the knee with varus deformity. OWHTO has been widely performed since good clinical results were first reported for innovative surgical procedures utilising biplanar osteotomy and locking plate fixation [1]. Special long locking plates, such as the TomoFix® (Johnson & Johnson, West Chester, PA), are known to provide good stability at the osteotomy site enabling early weightbearing after surgery with low risk of loss of correction or impaired bone union. The most proximal distal screw (screw 1) is usually fixed bicortically [1], but Staubli et al. reported delayed union in cases where all four distal screws were fixed monocortically. Therefore, there was variation depending on the surgeon’s preference for how many screws are fixed bicortically. In fact, some surgeons used bicortical fixation for all four distal screws [2, 3].

Recently, it was reported that the neurovascular bundle containing the deep peroneal nerve on the interosseous membrane of the leg is at potential risk of injury during drilling for distal screws on the locking plate [4–6]. The extended trajectories of the distal screws of the locking plates likely pass close to the neurovascular bundle located next to the lateral wall of the tibia, and neurovascular bundle injury can occur during drilling for bicortical fixation. The risk was relatively high for the four distal screws of both the TomoFix plate and the TriS® plate (Olympus Terumo Biomaterials Corp, Tokyo, Japan), the latter of which is a locking plate designed in Japan with a shape similar to that of the TomoFix. To avoid this risk, monocortical fixation of a few distal screws was recommended [5]. Nevertheless, the possibility of achieving biomechanical stability when monocortical fixation was performed in patients with low bone mineral density (BMD) is of great concern [7].

The purpose of this study was to analyse the biomechanical properties of OWHTO using a long locking plate with monocortical fixation for distal screws. The following research questions were therefore posed: (1) Do the biomechanical properties of the osteotomy site and plate differ between monocortical and bicortical fixation of three of the four distal screws? (2) Do the changes become more significantly different between low BMD bone compared with normal BMD bone? To answer these questions, three-dimensional (3D) OWHTO models were created with data of a locking plate system and computed tomography (CT) data from a representative subject, utilising the bicortical fixation for all four distal screws or monocortical fixation for screws 2, 3, and 4. Finite element analysis (FEA) was performed using FEA software to assess the stress distribution around the osteotomy site and the plate, and compared between the bicortical and monocortical fixation models on both normal- and low-BMD models. Furthermore, loss of correction was assessed by measuring the medial proximal tibial angle (MPTA) in 34 patients who underwent OWHTO in which monocortical and bicortical fixation were used for each of two distal screws at 1 month and 1 year after surgery.

## Materials and methods

**Fig. 1:**
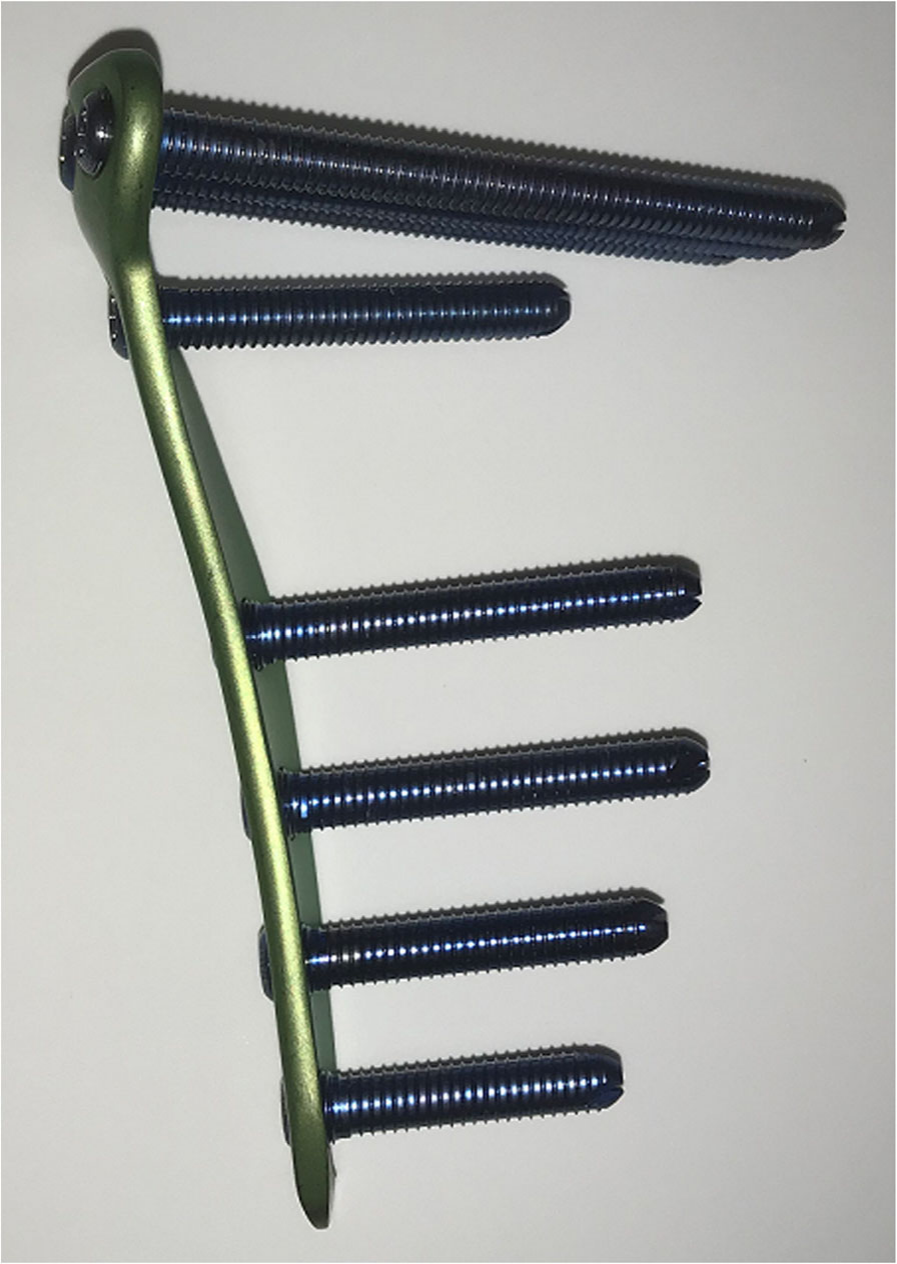
The TriS locking plate. A locking plate made in Japan with a shape similar to that of the TomoFix

CT data of a 62-year-old female patient with osteoarthritis of the knee (Kellgren-Lawrence grade 3, varus limb alignment with 4° hip-knee-ankle angle) who had undergone preoperative CT scanning at our institute and material data of a locking plate and screws were used to create 3D models of bone and fixation materials simulating OWHTO. The knee models were developed using Mechanical Finder 10.0 FEA software (Research Centre of Computational Mechanics Inc., Tokyo, Japan) and Metasequoia 4 3D modelling software (Tetraface Inc., Tokyo, Japan). In turn, the models were subjected to 10° valgus correction around the hinge axis. The hinge axis was defined as the axis passing anterior to posterior around the fibula head approximately 3.0 mm medial to the lateral tibial cortex. The oblique osteotomy surface started at the medial tibial cortex 35 mm distal to the joint line and was angled in the direction of the hinge axis. The TriS plate used in these models is an 11-cm long T-shaped locking plate with four screw holes (A, B, C, and D) for proximal fixation and another four holes (1, 2, 3, and 4) for distal fixation (Fig. 1). The thickness of the TriS plate was 3.1 mm. The TriS locking plate was placed as follows: (1) at the midpoint between the posterior of the proximal tibia and central screw of proximal holes of the plate just beside the proximal tibia; (2) all proximal screws were monocortical, even though a 60-mm screw was selected. In this study, the model was simplified by using a pin-shaped model instead of a screw. In addition, a previous report [8] described an artificial bone replacement material composed of beta-tricalcium phosphate (β-TCP) (a generic formulation of the bone void filler Osferion60; Olympus Terumo Biomaterials Corp, Tokyo, Japan), which was placed into the osteotomy gap so as not to stick out of the tibial cortex. However, the use of artificial bone is controversial. Accordingly, this study evaluated two different procedures, one with and one without artificial bone.

### Physical properties

Referencing the report on BMD status by Keyak et al. [9], the density distribution was calculated with the following formula using FEA software.

$$ \mathrm{BMD}\left[\mathrm{g}/{\mathrm{cm}}^3\right]=\left(\mathrm{CT}\ \mathrm{value}\ \left[\mathrm{H}.\mathrm{U}.\right]+1.4246\right)\times 0.001/1.058 $$

The calculation was done with normal density and on the assumption that a usual 1/4 density was representative of an osteoporosis patient. In contrast, the physical properties of Ti-6Al-4 V alloy, which is a constituent material of locking plates, were used as listed in the material properties library in the Mechanical Finder 10.0 Software. Physical properties of β-TCP, which is used as artificial bone, were provided by Olympus Terumo Biomaterials Corp. Actual values used are shown in Table 1.

**Table 1:** Physical parameters

	TriS locking plate	Artifical bone
Young’s modulus/GPa	108.9	0.3
Poisson’s ratio	0.3	0.4
Tensile strain/MPa	899.3	20
Compressive stress/MPa	824.7	20
Stress relaxation coefficient	0.1	0.1
Density mg/cm^3^	4430	1300
Fracture strength/μ	100,000	3000

### Constraint and loading conditions

A non-linear contact analysis was performed. The contact surface of artificial bone, cancellous bone, and cortical bone was analysed as the contact site, and the plate and cortical bone and cancellous bone were adherent to each other. The coefficient of friction at the contact surface was 0.45, and the stress relaxation coefficient was 0.1. It was assumed that a uniform distributed load of 375 N was applied to the tibial medial joint surface and 625 N to the lateral tibial joint surface, and the load was in the direction of the tibial axis. The axial compressive load was assumed to be 1000 N based on a previous study [10]. A 3D model produced with a length of about 230 mm from the tibial joint surface was fixed at 100 mm from the end of the model.

### Comparison of groups

The screw holes in the shaft were numbered 1, 2, 3, and 4 from the proximal to the distal side of the plate. As in the standard procedure, screw 1 was fixed bicortically [1].

1) Analysis A and Ap (osteoporosis): screws 2, 3, and 4 were fixed bicortically.

2) Analysis B and Bp (osteoporosis): screws 2, 3, and 4 fixed monocortically.

### Measuring parameters

For the calculation, the equivalent stress in the plate and bone at each part was mapped by colour tone. Fixed point observation of the following sites was performed in Analysis A, Ap, B, Bp, and the screw length that achieved necessary and sufficient fixed strength was considered.

**Fig. 2:**
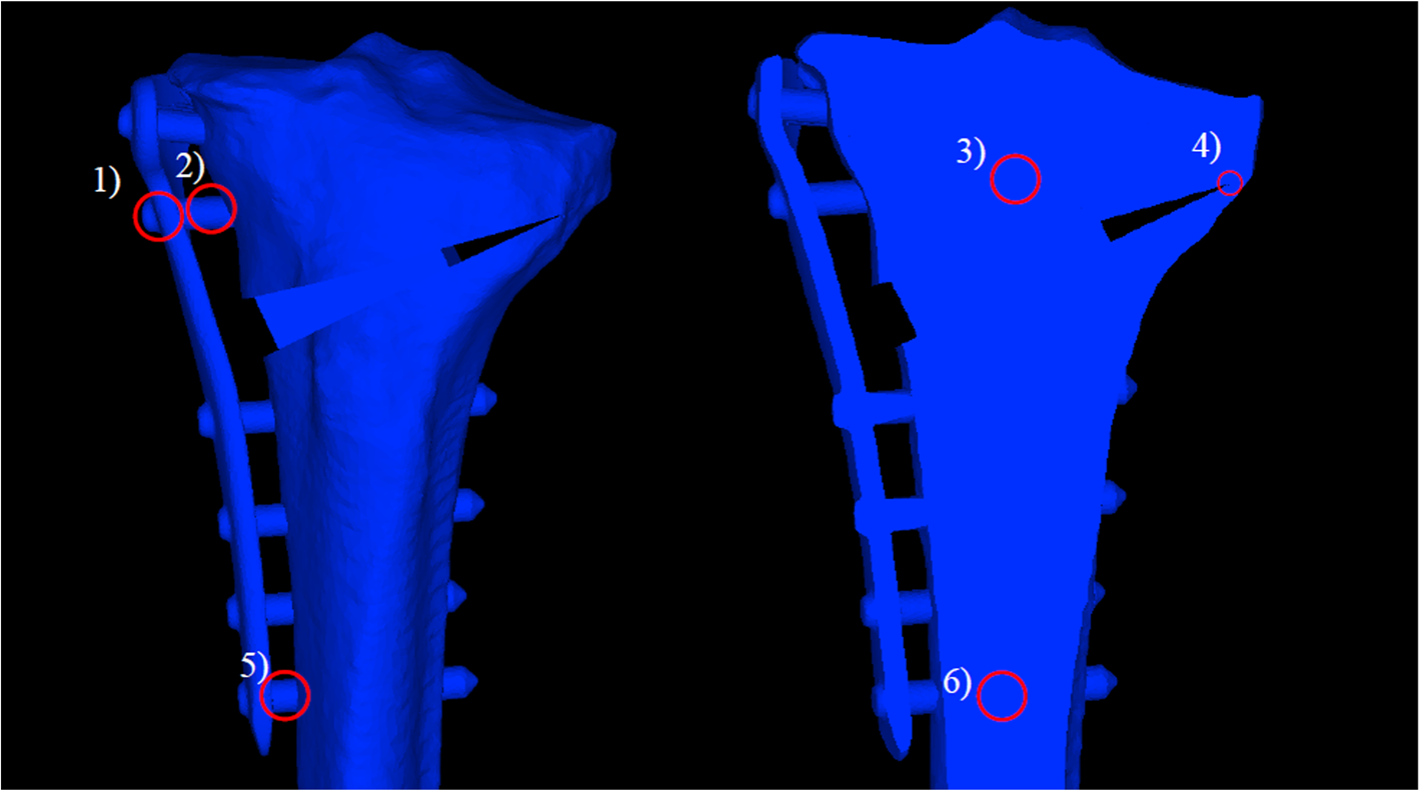
Measurement items (Analysis A). 1) D hole, 2) D screw proximal, 3) D screw distal in the bone, 4) lateral hinge, 5) screw 4 proximal, and 6) screw 4 distal in the bone

**Fig. 3:**
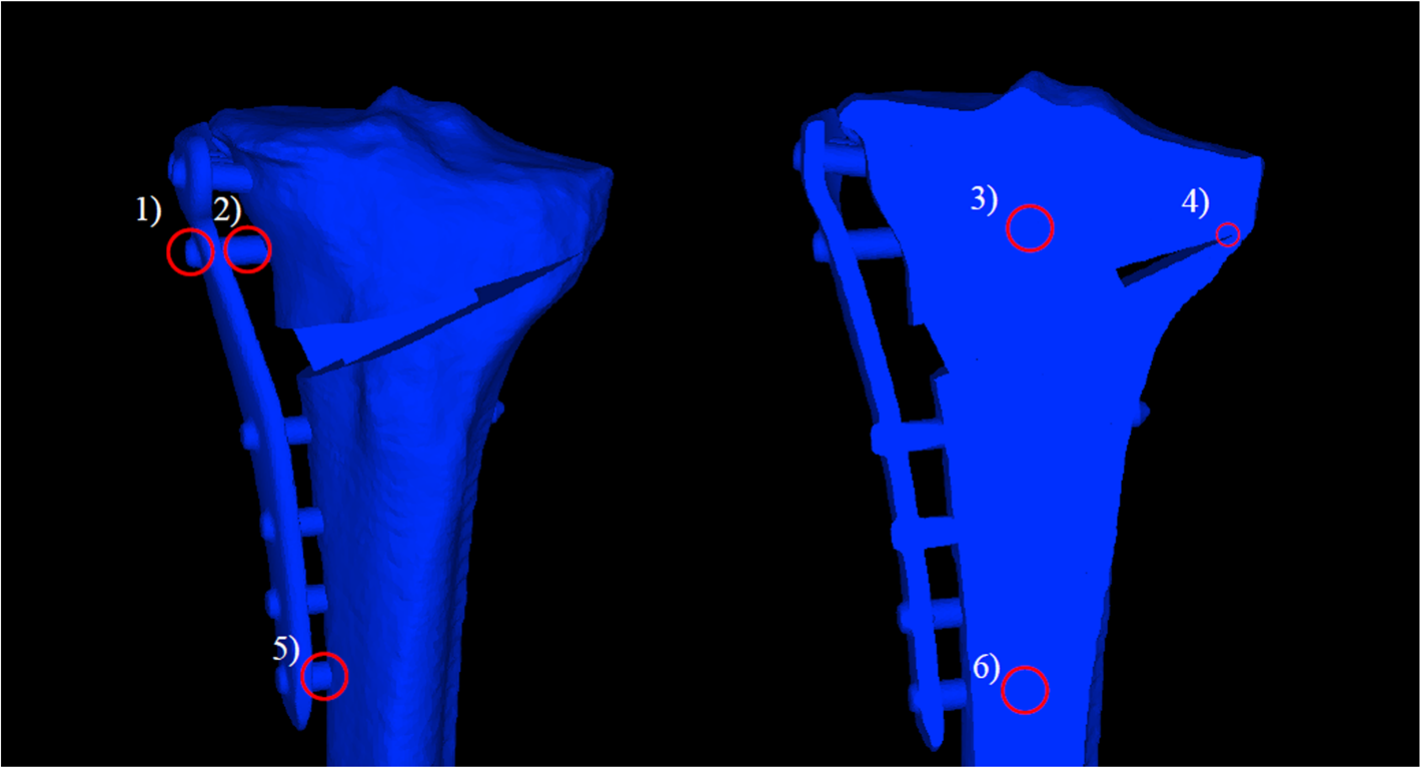
Measurement items (Analysis B). 1) D hole, 2) D screw proximal, 3) D screw distal in the bone, 4) lateral hinge, 5) screw 4 proximal, and 6) screw 4 distal in the bone

1) D hole; 2) D screw proximal; 3) D screw distal in the bone; 4) lateral hinge; 5) screw 4 proximal; and 6) screw 4 distal in the bone (Figs. 2 and 3).

For measurements, a sphere was placed in the region of interest (1–6 above), and the value in the sphere was measured. Measurements for the plate or the bone were made at equivalent stress values using the von Mises or the Drucker–Prager yield criterion.

### Assessment loss of correction in clinical practice

A total of 34 consecutive patients (19 women and 15 men; mean age at surgery, 57.3 [standard deviation, 9.6] years; mean body mass index at surgery, 26.8 [standard deviation, 4.0] %) who underwent OWHTO at our institution between 2017 and 2019 were enrolled. Inclusion criteria were (1) two of the four distal screws fixed in a monocortical fashion, and (2) full-length weightbearing radiographs obtained at both 1 month and 1 year after surgery. In contrast to the FEA method used in the present study, two of the four distal screws were fixed in a monocortical fashion because of the need to reduce the risk of neurovascular injury [4–6]. There was a limitation in the FEA itself as well as a risk of hinge fracture [11], and fixation of two distal screws was adopted as a compromise. The OWHTO procedure was performed using biplanar osteotomy and the artificial bone was inserted into the osteotomy gap. A TriS locking plate was used for osteosynthesis in all patients. MPTA was measured and compared between 1 month and 1 year after the surgery in order to assess the loss of correction.

### Statistical analysis

A paired *t*-test was used to compare early postoperative and one-year follow-up values for MPTA. JMP Pro (SAS, Cary, NC) was used for statistical analysis. *P* < 0.05 was considered statistically significant. To detect the effect size of Cohen’s d = 0.50 with 80% power (alpha = 0.05, two-tailed), G*Power (Version 3.1.; Kiel, Germany) suggested that 27 patients were needed for a paired samples *t*-test.

### Ethical approval and consent to participate

All procedures involving human participants were in accordance with the ethical standards of the 1964 Helsinki Declaration and its later amendments. The study was approved by the institutional review board of Tokyo Women’s Medical University (approval no.4713, March 28, 2018). Informed consent was obtained via an opt-out procedure.

## Results

**Fig. 4:**
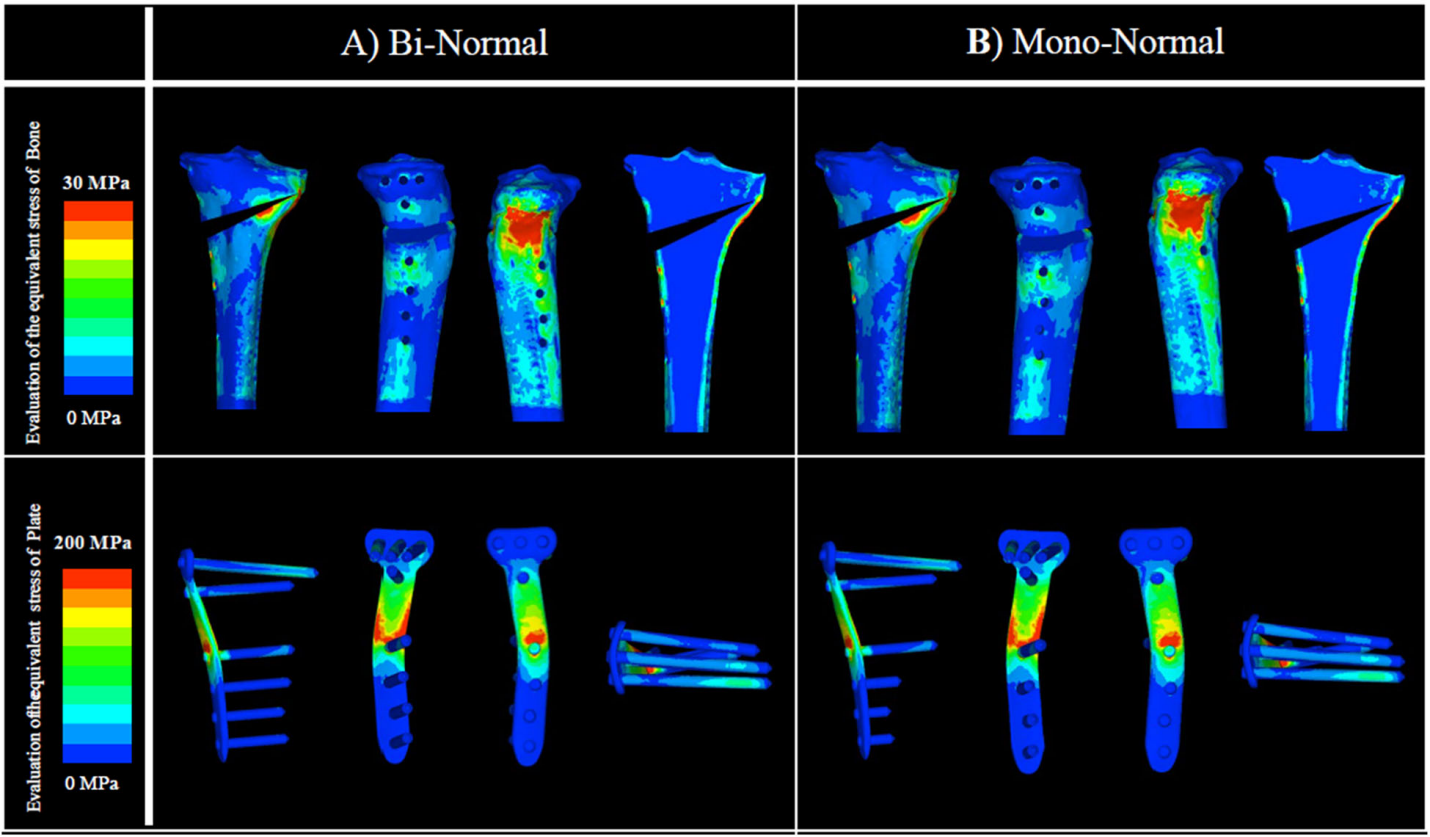
Evaluation of the equivalent stress in bone and plate: normal BMD without artificial bone. There was no clear difference between analyses A and B in 1) the D hole of the locking plate. Similarly, in 4) the lateral hinge, there was no clear difference between analyses A and B. BMD, bone mineral density

**Fig. 5:**
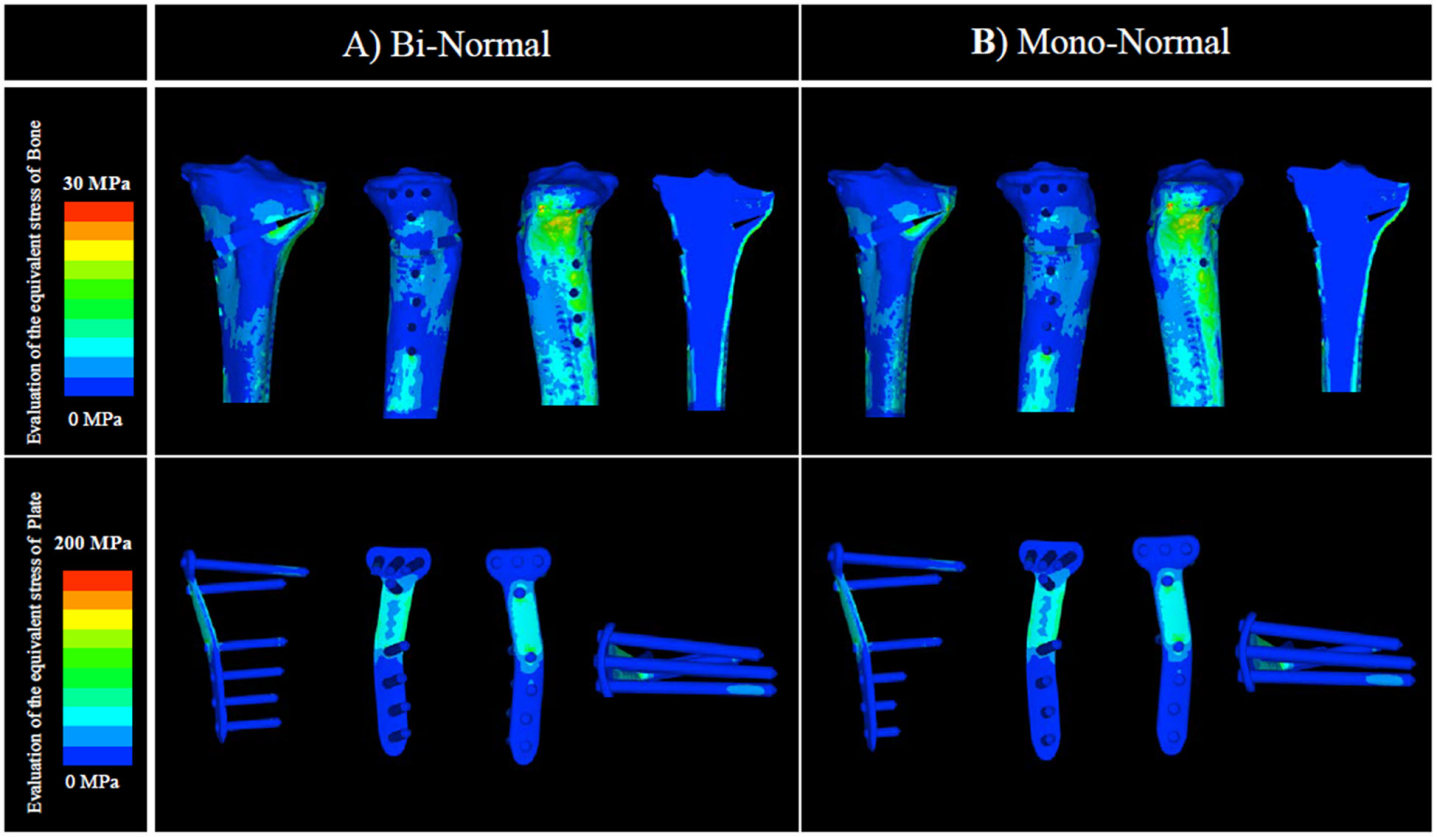
Evaluation of the equivalent stress in bone and plate: normal BMD with artificial bone. There was no clear difference between analyses A and B in 1) the D hole of the locking plate. Similarly, in 4) the lateral hinge, there was no clear difference between analyses A and B. BMD, bone mineral density

In the normal-BMD model, there was no clear difference between analysis A and analysis B, both with and without artificial bone, in the evaluation of the equivalent stress in the plate at 1) the D hole of the locking plate. Similarly, in the evaluation of the equivalent stress in bone at 4) the lateral hinge, the difference between analysis A and analysis B was not clear (Figs. 4 and 5; Tables 2 and 3).

**Table 2:** Parameters of equivalent stress without artificial bone

Equivalent stress/MPa	A	B	Ap	Bp
1) D hole	134	131	1000	962
2) D screw proximal	47.5	48.1	457.2	460.2
3) D screw distal in the bone	32.8	31.7	82.8	81.6
4) Lateral hinge*	36.3	36.2	17.6	17.4
5) Screw 4 proximal	37.9	29.6	65.4	47
6) Screw 4 distal in the bone	19.3	9.4	66.4	17

*Mean values are used to avoid dispersion of the computed tomography value

**Table 3:** Parameters of equivalent stress with artificial bone

Equivalent stress/MPa	A	B	Ap	Bp
1) D hole	104	101	331	313
2) D screw proximal	25	29.5	95	95.8
3) D screw distal in the bone	22.9	20.3	104	99.5
4) Lateral hinge*	20.7	21.6	6.6	6.8
5) Screw 4 proximal	33.4	33.5	63.6	41.5
6) Screw 4 distal in the bone	19.7	8.6	64.7	12.9

*Mean values are used to avoid dispersion of the computed tomography value

**Fig. 6:**
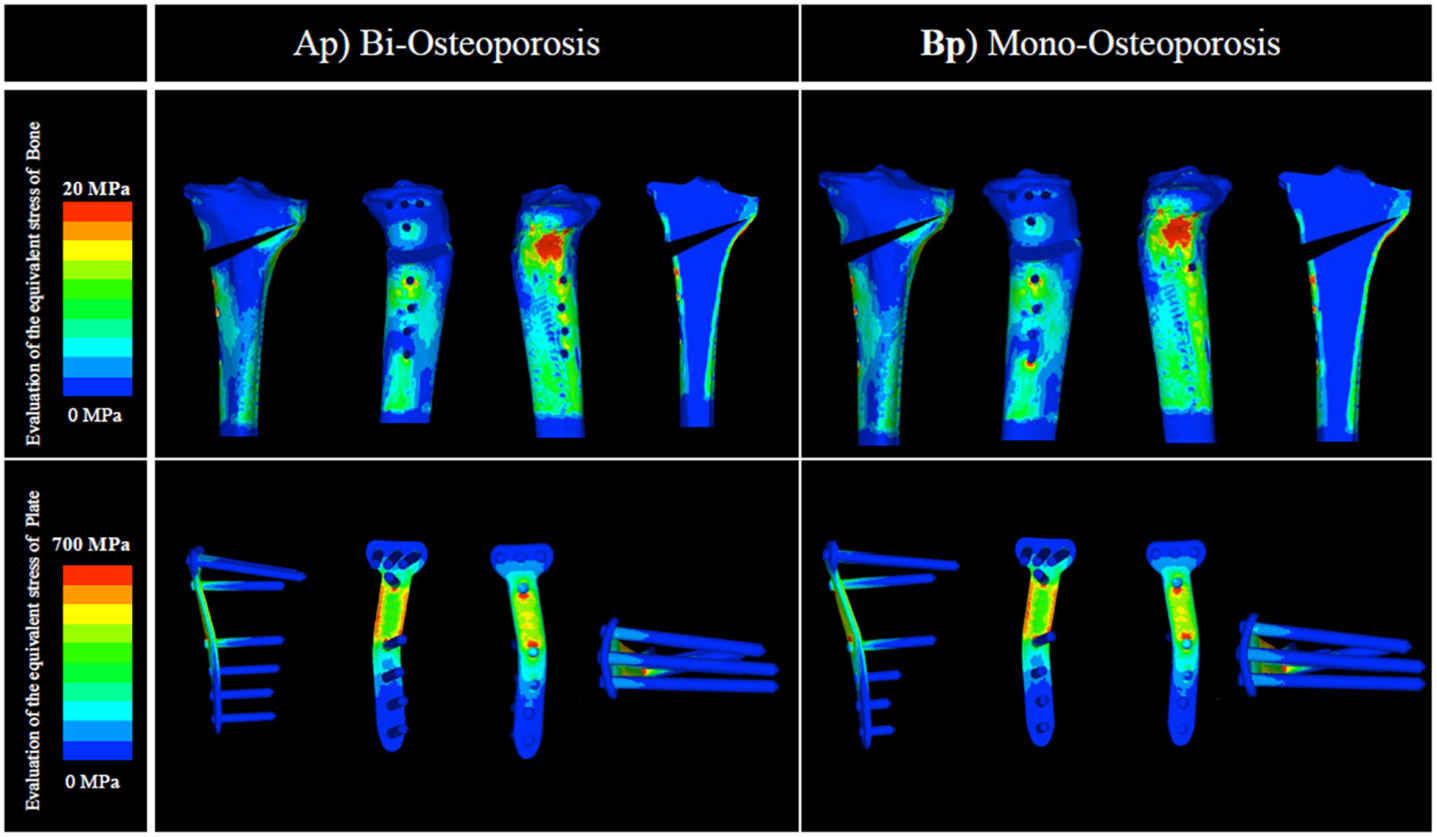
Evaluation of the equivalent stress in bone and plate: low BMD without artificial bone. There was no clear difference between analyses Ap and Bp in 1) the D hole of the locking plate. Similarly, in 4) the lateral hinge, no difference was observed between analyses Ap and Bp. BMD, bone mineral density

**Fig. 7:**
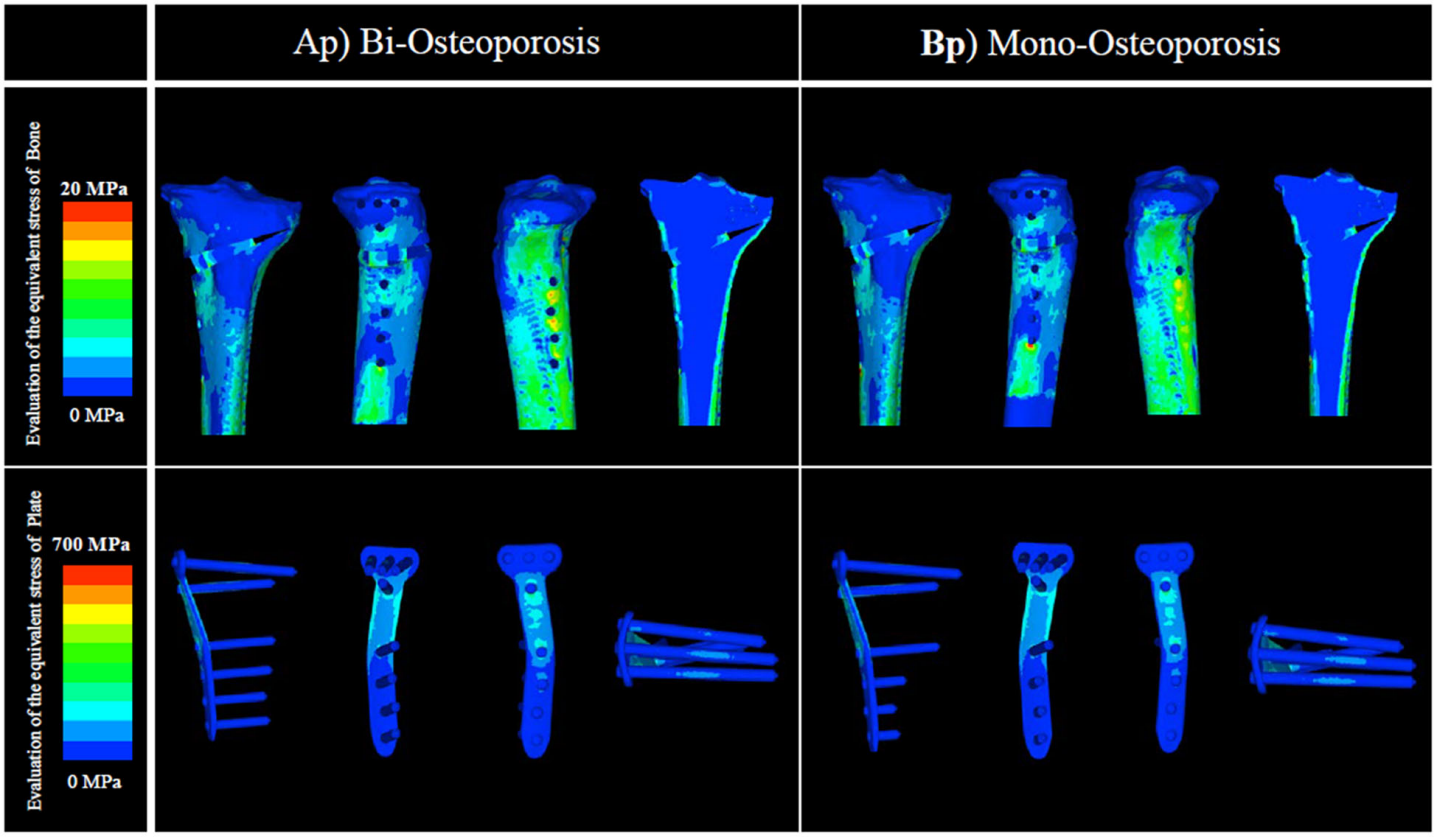
Evaluation of the equivalent stress in bone and plate: low BMD with artificial bone. There was no clear difference between analyses Ap and Bp in 1) the D hole of the locking plate. Similarly, in 4) the lateral hinge, no difference was observed between analyses Ap and Bp. BMD, bone mineral density

Moreover, in the osteoporosis model, there was no clear difference between analysis Ap and analysis Bp, both with and without artificial bone, in the evaluation of the equivalent stress in the plate at 1) the D hole of the locking plate; neither analysis Ap nor Bp indicated plate failure. For the evaluation of the equivalent stress in bone at 4) the lateral hinge, no difference was observed between analysis Ap and analysis Bp. The value of the equivalent stress in bone at 4) the lateral hinge indicated that lateral hinge fracture was highly unlikely (Figs. 6 and 7; Tables 2 and 3).

In the analysis comparing conditions with and without artificial bone, artificial bone reduced by approximately two thirds of the equivalent stress in the plate and the lateral hinge bone (Tables 2 and 3).

In the assessment of loss of correction by measuring postoperative MPTA, bone union at the osteotomy gap was confirmed in all the patients by radiography at 1 year after surgery. There were no significant differences in MPTA between the early postoperative period (90.7 ± 2.2) and 1-year follow-up (90.5 ± 2.2) (*p* = 0.98). Type 1 lateral hinge fracture (Takeuchi classification; type 1 fractures are stable, type 2 and type 3 fractures are unstable) [11] was observed in 4 cases, all of which were stable and had no obvious loss of correction. No other complications occurred, including deep peroneal nerve injury or breakage of the screws (Table 4).

**Table 4:** Demographic data and radiographic data in clinical practice

Age, years		57.3 ± 9.6
Female:Male		19:15
Body mass index, %		26.8 ± 4.0
MPTA, °	1 month	90.7 ± 2.2
	1 year	90.5 ± 2.2
Lateral hinge fracture		type1:4

In addition, a post hoc analysis of the correlation between early postoperative and one-year follow-up values for MPTA was performed. The statistical power was 0.88 with an effect size of 0.5, an alpha value of 0.05, and a sample size of 34.

## Discussion

The principal finding of this study was that, compared with bicortical fixation for all four distal screws, monocortical fixation for three distal screws (2, 3, and 4) of the locking plate did not impair the biomechanical stability of the OWHTO surgical simulation model with either normal or low BMD. The differences in the stress in the plate or lateral hinge between the two fixation methods were small. In the clinical cases, no loss of correction was observed prior to bone union in patients who underwent OWHTO using an artificial bone substitute and TriS locking plate according to the fixation method using two monocortical and two bicortical screws. Considering the advantage of avoiding risk of neurovascular bundle injury during bicortical drilling for distal locking screws, monocortical fixation for a few distal screws of the long locking plate is recommended.

The primary reason for choosing monocortical fixation for the distal screws was to avoid potential deep peroneal nerve injury [4]. The deep peroneal nerve is a branch of the common peroneal nerve and innervates mainly the tibialis anterior and the extensor hallucis longus [12]. Anatomically, the deep peroneal nerve arises from the common peroneal nerve and then descends with the anterior tibial artery and vein on the interosseous membrane [13]. Furthermore, the descending neurovascular bundle, containing the deep peroneal nerve, gradually approaches the tibia from the fibula [14]. Extended insertion trajectories of distal screws were likely to intersect with the interosseous membrane and potentially with the neurovascular bundle on its surface, and thus drilling with bicortical fixation posed a risk of neurovascular bundle injury [5]. Therefore, a safe method that avoids penetrating the lateral cortex for bicortical fixation, is recommended as long as good stability is ensured, and monocortical fixation of a few distal screws is such a method.

One of the findings that should be emphasised is that even with the low BMD model, the difference in stress distribution at the lateral hinge was not clear between bicortical fixation and monocortical fixation. Monocortical fixation could achieve sufficient stability even in Asians who have a low BMD and may be vulnerable to poor bone quality [7]. Further FEA analysis is warranted, given the differences in bone morphology between ethnicities.

In many previous studies, monocortical fixation of three of the four screws was considered popular with the use of the long locking plate during OWHTO [4, 15]. In contrast, bicortical fixation for all distal screws was seen in recent reports from Asia [2, 16]. The usefulness of bicortical fixation when using a locking plate is widely known [17], and so bicortical fixation is more frequently used. Bicortical fixation provides greater stability against torque [18]. Considering the findings of this study, monocortical fixation of two or three of the four distal screws may be advisable because it is a safer procedure with lower risk, such as neurovascular injury [5]. However, the continuity of the cortex at the lateral hinge was maintained in this simulation model. Lateral hinge fracture is a common postoperative complication of OWHTO [11] and has been reported to cause nonunion or loss of correction due to its unstable features [1, 19]. Nha et al. reported that plate breakage was associated with the presence of lateral cortical hinge fracture [20]. Furthermore, lateral hinge fracture is sometimes overlooked during surgery [21]. Lee et al. reported that most lateral hinge fractures were not identified on postoperative radiographs [22]. Therefore, some of the screws should be fixed bicortically to secure rotational stability in case lateral hinge fractures occurs. This is the reason why two of the four distal screws were fixed bicortically in the clinical cases in this study, despite the FEA model showing that stability was secured by using one bicortical and three monocortical fixation screws. The clinical study revealed no loss of correction prior to bone union. Thus, we suggest the use of two monocortical fixation screws as the most well-balanced method for securing stability, even in the case of hinge fracture and for reducing the risk of deep peroneal nerve injury, which is more likely with the use of distal two screws [5].

The use of artificial bone is controversial. Floerkemeier et al. reported favourable mid-term results with the use of TomoFix without artificial bone [23]. In contrast, Nha et al. reported that artificial bone was inserted to minimise postoperative loss of correction when the osteotomy gap was more than 12 mm [20]. Takeuchi et al. reported that they had prepared several artificial bones with heights of 7.5, 10, 12.5, and 15 mm to insert at the osteotomy site [8]. A previous biomechanical study reported that the use of artificial bone and TomoFix would improve stability at the osteotomy site [24]. Our study evaluated two different procedures, that is, with or without artificial bone. In addition, our findings revealed that the equivalent stress in the plate and at the lateral hinge were reduced by inserting artificial bone into the osteotomy gap; the difference was more remarkable in the osteoporosis model. Thus, the use of artificial bone may be desirable for relatively elderly patients with impaired bone strength.

This study has several limitations. First, the findings may not be applicable to all patients because CT data were derived from only one patient with osteoarthritis of the knee. However, because the study also investigated a model with significantly low BMD, the range of this study could be expected to apply to most categories of patients. Second, the 3D models used in this study were simplified to avoid excessive complexity in calculation. Therefore, some of the soft tissues, such as cartilage and meniscus and fibula were not accounted for. In addition, the 3D models were simplified with the use of pins instead of screws. This modification has also been used in past OWHTO studies with FEA [25]. Third, the osteotomy was not biplanar but monoplanar. These limitations of the 3D models may impair the mechanical properties compared with more realistic models. Nevertheless, this study focused on the differences in mechanical properties between the two fixation methods and found minimal differences in the models. Furthermore, biplanar osteotomy is considered to increase the stability of the osteotomy site. Therefore, only a slight significant difference in stability is expected between monocortical fixation and bicortical fixation. Fourth, only vertical loading was applied to the models, so the joint kinetics during motion were not reproduced. Fifth, only one plate system was evaluated. Different results might be obtained when using different plate systems.

## Conclusions

In OWHTO with a long locking plate, simulation using FEA was performed to compare the stress distribution when three of the four distal screws were fixed monocortically or bicortically. In both normal-BMD and low-BMD (osteoporosis) models, there was no clear difference in stress distribution at the osteotomy site including the lateral hinge. This suggests that the change in biomechanical strength was small even with monocortical fixation of up three of the four distal screws of the long locking plate. Therefore, this fixation method is advisable to avoid risk of neurovascular bundle injury during bicortical drilling without loss of stability. In actual surgery, the number of distal bicortical screws should be reduced based on the patient’s condition, considering the risk of lateral hinge fracture and unexpected surgical complications. Accordingly, the use of two bicortical and two monocortical fixation screws for the four distal screws is advisable for ensuring stability even with occult hinge fracture and reducing the risk of neurovascular injury during bicortical drilling.

## Data Availability

The datasets used and/or analysed during the current study are available from the corresponding author on reasonable request.

## Abbreviations

OWHTO: open-wedge high tibial osteotomy
BMD: bone mineral density
CT: computed tomography
FEA: finite element analysis
β-TCP: beta-tricalcium phosphate
MPTA: medial proximal tibial angle
